# Prognostic impact of CD123 overexpression on the outcome of pediatric acute myeloid leukemia

**DOI:** 10.3389/fonc.2026.1879782

**Published:** 2026-08-21

**Authors:** Mona Mohammed, Iman Sidhom, Nahla El-Sharkawy, Mahmoud Hammad, Maram Salama, Sonia Soliman, Dina Yassin, Sherine Salem, Nayera Hamdy, Amr Elnashar, Sherif Abouelnaga, Sonia Ahmed, Ahmed Elhemaly, Alaa Elhaddad

**Affiliations:** 1 Department of Pediatric Oncology, Children’s Cancer Hospital Egypt, Cairo, Egypt; 2 Department of Pediatric Oncology, National Cancer Institute, Cairo University, and Children’s Cancer Hospital Egypt, Cairo, Egypt; 3 Department of Clinical Pathology Children’s Cancer Hospital Egypt and National Cancer Institute, Cairo University, Cairo, Egypt; 4 Department of Pediatric Oncology, National Cancer Institute, Cairo University, Cairo, Egypt; 5 Department of Clinical Research, Children’s Cancer Hospital Egypt, Cairo, Egypt

**Keywords:** acute myeloid leukemia, CD123 expression, CD123 immunophenotyping, IL-3 receptor α chain, pediatrics

## Abstract

**Background and aims:**

Despite advances in acute myeloid leukemia (AML) therapy, outcomes remain poor. CD123 overexpression influences proliferation and enhanced survival of leukemic cells. Its impact on the outcome is still debatable. This study aimed to assess CD123 overexpression frequency in pediatric AML, its relation with disease features, and its impact on outcomes.

**Methods:**

This retrospective study included pediatric patients with *de novo* AML (≤18 years) treated with a protocol adapted from COGAAML 1031. CD123 expression ≥20% on AML blasts assessed by multicolor flow cytometry was considered positive. Survival outcomes were analyzed using Kaplan–Meier estimates, with cumulative incidence of relapse in the competing risk setting compared by Gray’s test, and multivariable associations assessed through Cox regression.

**Results:**

Among 403 patients, 141 (35%) were CD123^+ve^, showing a median expression level of 46%. CD123^+ve^ was significantly associated with higher white blood cell count (*P = 0.003*), percentage of bone marrow blasts (*P = 0.02*), M4/M5 FAB subtypes (*P < 0.001), FLT3-ITD (P = 0.001)*, and inversely related with t(8;21) *(P = 0.02)*. Conversely, CD123^+ve^ did not show significant association with age, sex, initial risk and other genetic abnormalities and did not influence the response to induction therapy, whether assessed morphologically or by minimal residual disease levels. No significant differences in survival outcomes were observed between CD123^+ve^ and CD123^−ve^ patients in both univariate and multivariable analyses. The 3-year EFS was 45.3% (95% CI; 37.8-54.3) for CD123^+ve^ compared to 42.2% (95% CI; 36.6-48.7) in CD123^-ve^ patients (*P = 0.5*) and the 3-year OS was 51.7% (95% CI; 44.1-60.7) versus 49% (95% CI; 43.2-55.5), respectively (*P = 0.6*). The cumulative incidence of relapse at 3 years for CD123^+ve^ was 24.1% (95% CI; 17.4-31.5), comparable to 23.7% (95% CI; 18.7-29) for CD123^-ve^ patients (*P = 0.9*).

**Conclusions:**

The association of CD123^+ve^ with initial higher disease burden and FLT3-ITD, implies that it may mark a biologically distinct and more aggressive leukemia subtype. Although its prognostic impact remains unclear, its high incidence supports its potential as a therapeutic target, necessitating further large-scale studies.

## 1 Introduction

Acute myeloid leukemia (AML) is a clonal disorder of hematopoietic stem cell driven by abnormal proliferation and impaired differentiation, resulting in the accumulation of myeloblasts. It is the second most prevalent hematologic malignancy in children, accounting for approximately 15-20% of acute leukemia cases (1). Pediatric AML poses a significant clinical challenge due to its variable response to treatment and relatively low survival rates. Even though contemporary induction chemotherapy achieves high rates of complete remission (CR), the overall survival (OS) still ranges from 60-70% (2, 3). Furthermore, despite advances in supportive care and risk-adapted treatment protocols, event-free survival (EFS) remains around 50–60%, with relapse constituting a major cause of treatment failure (4). Understanding the mechanisms that govern the formation and persistence of leukemic stem cells (LSCs) is crucial for identifying novel therapeutic opportunities (5). CD123, also known as the interleukin-3 receptor (IL-3Rα), is a cell membrane glycoprotein composed of a distinct α chain paired with a β subunit. This β subunit is shared with the receptors for interleukin-5 (IL-5) and granulocyte-macrophage colony-stimulating factor (GM-CSF), collectively known as the beta common (βc) family of membrane receptors. This βc family of membrane receptors regulates the growth, proliferation, survival, and differentiation of hematopoietic cells, as well as immunity and inflammatory response (6, 7). Once IL-3 binds to CD123, the βc subunit (CD131) is recruited to form a high-affinity receptor. Then, this receptor initiates intracellular downstream signaling pathways that in turn activate the *JAK/STAT, RAS-MAPK*, and phosphatidylinositol 3-kinase pathways promoting cell proliferation and survival (8). Most notably, CD123 is frequently overexpressed on both LSCs and more differentiated leukemic blasts, while being expressed at much lower levels by normal myeloid progenitors (9, 10). Thus, CD123 has emerged as a promising therapeutic target, with the potential to both reduce tumor burden and decrease residual disease for sustained therapeutic responses (11). CD123 is prominently expressed in various hematological malignancies, including AML, acute lymphoblastic leukemia (ALL), blastic plasmacytoid dendritic cell neoplasm (BPDCN), myelodysplastic syndrome (MDS), chronic myeloid leukemia (CML), hairy cell leukemia, and Hodgkin’s lymphoma (6). Numerous studies have demonstrated that CD123 is expressed in 45–95% of AML cases, highlighting its critical role in characterizing leukemic blasts and LSCs. Furthermore, it has shown diagnostic significance in AML (12). Flow cytometry analyses have revealed that AML LSCs express CD123, while it is absent in normal hematopoietic stem cells (HSCs). This aberrant expression is a defining feature of AML LSCs suggesting its potential as a therapeutic target (13, 14). In some of clinical setting of AML, the over expression of CD123 is associated with lower complete remission rate and reduced 5-year survival (6, 7). However, most of these studies have focused on adult populations, leaving a significant knowledge gap in pediatric patients. The frequency and prognostic significance of CD123 expression in pediatric AML remains unclear and requires further investigation. In this study, we aimed to assess the frequency and level of expression of CD 123 in pediatric AML patients, and its relation to clinico-pathological characteristics as well as the impact of CD123 overexpression on treatment response and outcome.

## 2 Patients and methods

### 2.1 Study design and population

This is a retrospective observational study, which included pediatric patients, ≤18 years old, diagnosed with *de-novo* AML and treated at Children’s Cancer Hospital Egypt-57357 (CCHE-57357) from January 2018 to December 2021, with a minimum two-year follow-up period from the end of treatment. Patients were excluded if they had acute promyelocytic leukemia, therapy-related AML, relapsed AML, isolated extra-medullary myeloid sarcoma, myelodysplastic syndrome, AML patients with Down syndrome or Fanconi anemia, or if CD123 expression data were missing.

### 2.2 Risk stratification and treatment

All patients were treated using the CCHE-57357 AML protocol adapted from the COG AAML1031 protocol (clinicaltrials.gov NCT01371981) (15). Patients were initially stratified based on cytogenetics and molecular abnormalities into three risk groups:

#### 2.2.1 Low-risk

Patients with favorable cytogenetics including core binding factor (CBF)](t(8;21) (q22;q22.1); *RUNX1::RUNX1T1*, inv (16) (p13.1;q22) or t(16;16) (p13.1;q22); *CBFB::MYH11*[, as well as nucleophosmin 1 (*NPM1*) and *CEBPA* mutations.

#### 2.2.2 High-risk

Unfavorable cytogenetics as monosomy 7, monosomy 5, complex karyotype (more than three chromosomal aberrations), and FLT3 internal tandem duplications (*FLT3-ITD*) with a high allelic ratio (> 0.4)) were assigned to the HR group.

#### 2.2.3 Intermediate risk

Patients lacking both favorable and unfavorable cytogenetics were categorized as IR at diagnosis.

After the first induction, IR patients were reclassified into HR and LR categories based on minimal residual disease (MRD) assessed via multiparametric flow cytometry (MPFC), with LR defined as MRD <0.1% (16). All patients received four cycles of chemotherapy. Only high-risk patients in CR were offered allogeneic hematopoietic stem cell transplantation if a matched sibling donor was available (detailed treatment is shown in **Supplementary Figure 1**).

### 2.3 Treatment response definitions

Complete remission (CR): defined as < 5% blasts by morphology and no extramedullary disease. Refractory disease was defined as ≥5% BM blasts, morphologically or by proven extramedullary disease at the end of induction therapy. Relapse was defined as the reappearance of blasts in peripheral blood (PB) or bone marrow (BM) or the development of extramedullary disease after initial morphologic CR.

### 2.4 Ethical aspects

The institutional review board approved the study, and patient data confidentiality was strictly maintained.

### 2.5 Data collection

We reviewed the patients’ electronic medical records, and collected the following data: age at presentation, white blood cell count (WBC), peripheral and bone marrow (BM) blasts, FAB classification, cytogenetic and molecular abnormalities, CD123 expression via flow cytometry, response to treatment following induction I and II chemotherapy courses assessed morphologically and by MRD via flow cytometry, date of relapse and death, the status and date of the last follow-up. All patients included in the study underwent the same standard molecular diagnostic workup at diagnosis including assessment of *FLT3-ITD*, *RUNX1::RUNX1T1, CBFB::MYH11, NPM1* and *CEBPA* mutations.

### 2.6 Immunophenotyping analysis

CD123 immunophenotyping was performed on the initial BM samples at diagnosis using a PE-conjugated anti-CD123 antibody (SSDCLY107D2; Beckman Coulter). Immunophenotyping was conducted utilizing MPFC. A total of 20,000 events per sample were recorded. The blast gate was defined based on CD45 dim expression and side-scatter characteristics quantified as a percentage of total gated events. The mean fluorescence intensity (MFI) of leukemic blasts was adjusted for background fluorescence using residual normal lymphocytes in each sample as a negative control. The gating strategy for CD123 expression involved the creation of dot plots for the acquisition sample tubes; one plot displayed Forward Scatter (FSc) on the X-axis versus Side Scatter (SSc) on the Y-axis to identify the size and granularity of the collected cells and to exclude debris and dead cells. The second plot displayed the antibody marker CD45 on the X-axis versus SSc on the Y-axis for gating the dim CD45 malignant clone. Patterns and analysis of the sample tubes were performed as follows: cursors were set to differentiate between positive and negative populations depending on the isotype control plot analysis. The marker set in the negative control plot was copied onto the histogram into four quadrants. Analysis of the population in each quadrant was as follows: the lower left quadrant, double negative for both markers; the lower right quadrant, positive for the X-axis marker only; the upper-left quadrant, positive for the Y-axis marker only; and the upper right quadrant, positive for both markers (**Supplementary Figures 2A–C**).

For the analysis of CD123 expression, percentage expression and MFI were assessed. CD123 positivity (CD123^+ve^) in this study was defined as expression ≥20% in accordance with thresholds commonly applied in flow cytometry reporting and consistent with previously published studies evaluating CD123 expression in acute myeloid leukemia (11).

Detection of MRD post-induction I and induction II were assessed by MPFC at a cut-off 0.1% according to the COG AAML 1031 protocol (16). MRD was assessed in all patients after each treatment cycle using MFC on the Navios EX (Beckman Coulter) according to the European LeukemiaNet (ELN) proposed consensus with 8–10 color monoclonal antibody panels (17). Two approaches were applied: leukemia-associated immunophenotype (LAIP) and different-from-normal (DFN). MRD positivity was defined by the presence of any LAIP population or any immunophenotypic deviation from normal patterns (18).

### 2.7 Statistical methodology

SPSS (statistical package for social sciences) version 27.0 was used for data management and analysis. Mean ± standard deviation described quantitative variables and median with range when appropriate. Number and percentages described qualitative data and Chi-square or/Fisher exact tested proportion independence. Parametric and non-parametric t tests compared two independent groups. Overall survival (OS) was calculated from the date of diagnosis to the date of death or last contact. Event-free survival (EFS) was measured from the date of diagnosis to the date of earliest event including relapse, second malignancy, or death due to any cause (19). Kaplan Meier method was used to estimate survival and log rank test to compare survival curves. Variables considered clinically relevant were entered into a multivariable Cox proportional hazards regression model to identify independent predictors of OS and EFS. Adjusted hazard ratios with 95% confidence intervals (CIs) were reported. Cumulative incidence of relapse (CIR) was calculated to estimate relapse incidence in a competing risk setting with Gray’s test and was conducted utilizing the R statistical software (R version 4.4.1). P values are always two-tailed and significant at < 0.05 level. Missing data were handled using available-case analysis. Patients with missing values for a given variable were excluded only from analyses involving that variable, while all other available data were retained.

## 3 Results

### 3.1 Patient’s characteristics

This study included 403 AML patients with a median age at diagnosis of 7.3 years (range 0.12–18 year), male to female ratio 1.35:1. The median value for WBCs was 16x10^3/^mcl (range 0.9-417) and the most common cytogenetic abnormalities were t(8;21) in 20.7%, KMT2A rearrangements (*KMT2A-r*) in 11.9% and inversion 16 in 9.9% of the patients. CD123^+ve^, defined as ≥20% expression on blast cells using MPFC, was found in 141 (35%) patients. Among CD123^+ve^ cases, the median expression level was 46% (**Supplementary Figure 1**). A total of 136 (33.7%) patients were initially classified as LR, 55 (13.6%) patients as HR, and 212 (52.6%) patients, with neither favorable nor unfavorable molecular or cytogenetic abnormalities, were categorized as IR. Following induction I in the IR group, twelve patients with MRD <0.1% were recategorized as low risk, 182 patients with MRD ≥ 0.1% as high-risk and 18 patients died during induction I before BM assessment. Thirty patients (7.4% of the whole cohort, 19.6% of the high-risk group) underwent allo-HSCT in first complete remission from a matched sibling donor using Busulfan-based conditioning.

### 3.2 CD123 expression and its association with initial patients’ characteristics

CD123^+ve^ was significantly associated with elevated WBCs (*P = 0.003*), higher percentage of BM blasts (*P = 0.02*), and FAB M4/M5 subtypes (*P < 0.001)*. In molecular and cytogenetic analysis, it was significantly associated with *FLT3-ITD* (allelic ratio >0.4) *(P = 0.001)*, while patients with *t(8;21)* showed significantly lower frequency of CD123^+ve^ expression *(P = 0.02).* No significant association was found with age, sex, peripheral blasts, *inv*16 or t (16;16)*, KMT2A-r*, complex karyotype, *NPM/CEPBA* mutations and monosomy 7. Although the high-risk group had a higher proportion of positive CD123 than the low- and intermediate-risk groups, this difference did not reach statistical significance (*P = 0.1).* No significant association was observed between CD123 expression and receipt of HSCT in first complete remission (*P = 0.42*) (**Table 1**).

**Table 1:** Demographic, clinical and treatment characteristics according to CD123 expression.

Clinical and treatment characteristics:	Patients number	CD123 expression (cut off 20%) N (%)		P value*
	Total N = 403	Positive 141(35%)	Negative 262 (65%)	
Age in years:				0.9
Mean ± SD		7.85 ± 4.84	7.86 ± 5.36	
Age:				0.7
<10 years	243	83 (34.2%)	160 (65.8%)	
≥ 10 years	160	58 (36.2%)	102 (63.8%)	
Sex:				0.09
Male	232	73 (31.5)	159 (68.5)	
Female	171	68 (39.8)	103 (60.2)	
WBCs				0.003
Median (range)		26.0 (0.9 – 417)	14.2 (0.9 – 398.0)	
Peripheral blasts %**				0.1
Median (range)		38.0 (0 – 98.0)	28.0 (0 – 99.0)	
Bone marrow blasts %				0.02
Median (range)		70.0 (5.0 – 98.0)	60.0 (9.0 – 97.0)	
FAB classification:				<0.001
M0	28	10 (35.7)	18 (64.3)	
M1	77	26 (33.8)	51 (66.2)	
M2	112	32 (28.6)	80 (71.4)	
M4	101	48 (47.5)	53 (52.5)	
M5	35	20 (57.1)	15 (42.9)	
M6	2	0 (0)	2 (100)	
M7	48	5 (10.4)	43 (89.6)	
t(8;21) **				0.027
Yes	83	20 (24.1)	63 (75.9)	
No	318	120 (37.7)	198 (62.3)	
t(16;16), inv16**				0.2
Yes	40	17 (42.5)	23 (57.5)	
No	361	123 (34.1)	238 (65.9)	
NPM1/CEBPA˚				0.3
Yes	12	6 (50)	6 (50)	
No	380	130 (34.2)	250 (65.8)	
FLT3-ITD ˚˚				0.001
Yes	22	15 (68.2)	7 (31.8)	
No	379	124 (32.7)	255 (67.3)	
KMT2A-r				0.5
Yes	48	19 (39.6)	29 (60.4)	
No	355	122 (34.4)	233 (65.6)	
Monosomy 7				0.6
Yes	18	5 (27.8)	13 (72.7)	
No	385	136 (35.3)	249 (64.7)	
Complex Karyotyping				0.6
Yes	19	8 (42.1)	11 (57.9)	
No	384	133 (34.6)	251 (65.4)	
WT1^∞^				0.7
Yes	210	75 (35.7)	135 (64.3)	
No	183	62 (33.9)	121 (66.1)	
Initial risk:				0.1
LR	136	44 (32.4)	92 (67.6)	
IR	212	71 (33.5)	141 (66.5)	
HR	55	26 (47.3)	29 (52.7)	
HSCT_CR1				0.4
Yes	30	13 (43.3%)	17 (56.7%)	
No	373	128 (34.3%)	245 (65.7%)	

*Significant P-value < 0.05. LR, low risk; IR, intermediate risk; HR, high risk. HSCT_CR1: Hematopoietic stem cell transplant_complete remission. **One patient is missing in each group. ˚Eleven patients do not have available CEBPA and NPM (5 from positive expression group and 6 from negative expression group). ˚˚2 patients are missing from positive expression group. ^∞^Ten patients are missing WT1 (4 from positive expression group and 6 from negative expression group).

### 3.3 CD123 expression in relation to treatment response

#### 3.3.1 Induction I

**Figure 1:**
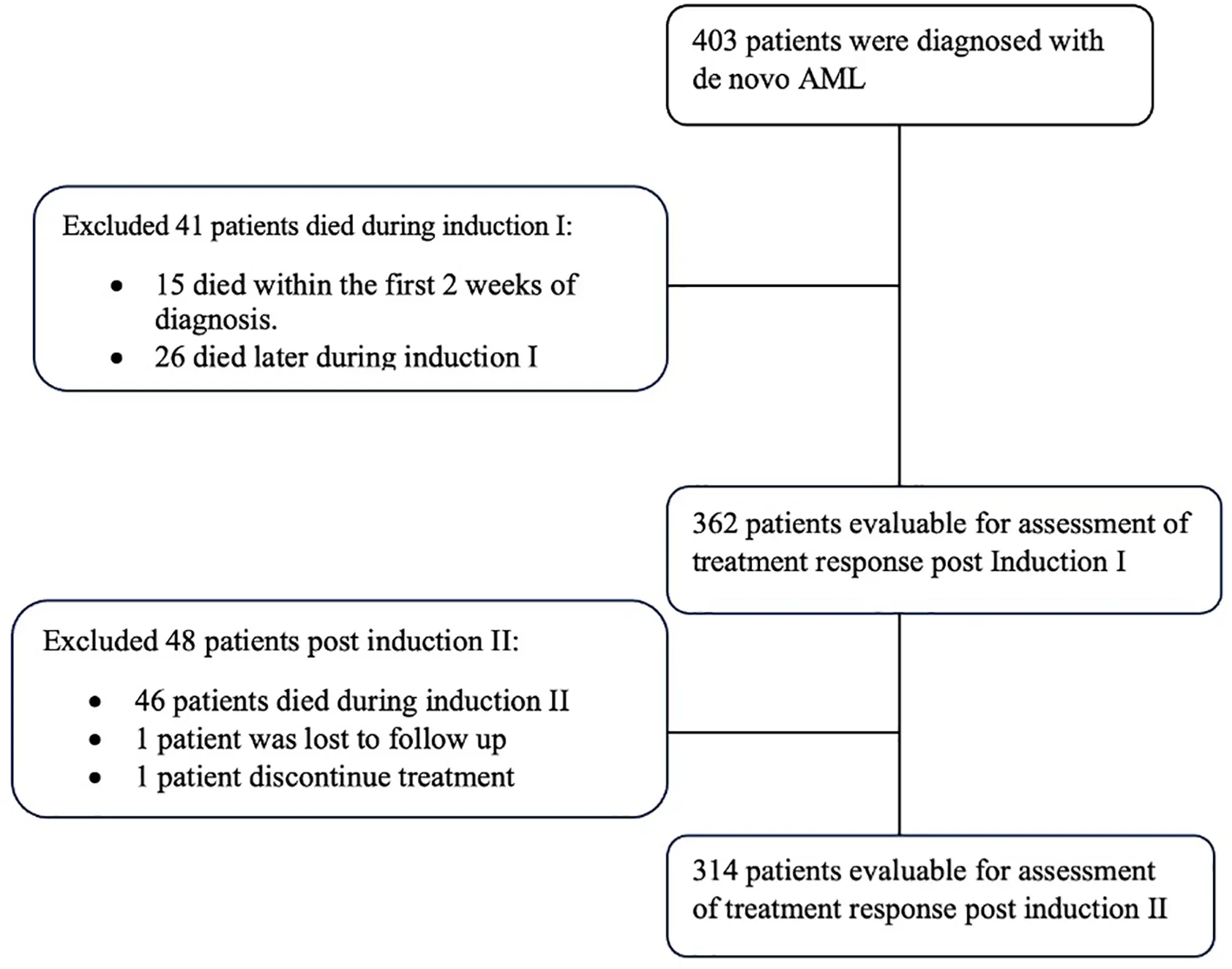
Flow diagram of pediatric patients with *de novo* AML enrolled in the study.

Forty-one patients died during induction I and 362 patients were eligible for response assessment. Among 127 patients who were CD123^+ve^, 63 (49.6%) patients achieved CR (blasts <5%) compared with 123 patients of the 235 who were negative for CD123 expression (52.3%) *(P = 0.6).* Based on MRD assessment post-induction I, five patients of the CD123^+ve^ (3.9%) as well as five patients with CD123^-ve^ expression (2%) achieved MRD < 0.1% *(P = 0.3)* (**Table 2**, **Figure 1**).

**Table 2:** Distribution of CD123^+ve^ in relation to treatment response post-induction I and II.

Treatment response	Total patients	CD123 expression		P value*
	Total no. (%)	Positive (%)	Negative (%)	
Response post induction I:	No=362			
Morphological response:		No= 127 (35%)	No= 235 (65%)	0.6
CR	186 (51.4)	63 (49.6%)	123 (52.3%)	
No CR	176 (48.6)	64 (50.4%)	112 (47.7%)	
MRD:				0.3
<0.1%	10 (2.8)	5 (3.9%)	5 (2%)	
≥0.1%	352 (97.2)	122 (96.1%)	230 (98%)	
Response post induction II:	No=314			
Morphological response:		No=112 (35.7)	No= 202 (64.3)	0.5
CR	291 (92.7)	102 (91)	189 (93.5%)	
Refractory	22 (7)	10 (9)	12 (6)	
Relapse	1 (0.3)	--	1 (0.5)	
MRD post induction II:				0.4
<0.1%	131 (41.7)	50 (44.6)	81 (40)	
≥0.1%	183 (58.3)	62 (55.4)	121 (60)	

*Significant P-value < 0.05. CR, complete remission; MRD, minimal residual disease.

#### 3.3.2 Induction II

Three hundred and forteen patients were eligible for assessment of treatment response post induction II (46 patients died during induction II before BM or MRD assessment, one patient lost follow up post induction I and treatment had been discontinued for one patient post induction I due to significant morbidity). Of 112 patients with CD123^+ve^, 102 patients (91%) were in CR and 10 patients (9%) were refractory. Among 202 patients who were CD123^-ve^, 189 (93.5%) were in CR, 12 (6%) were refractory, and one patient (0.5%) relapsed. These differences were statistically not significant *(P = 0.5).* Regarding MRD assessment, 50 patients (44.6%) of the CD123^+ve^ and 81 patients (40%) of CD123^-ve^ achieved MRD levels below 0.1% *(P = 0.4)* (**Table 2**, **Figure 1**).

### 3.4 Survival analysis

**Figure 2:**
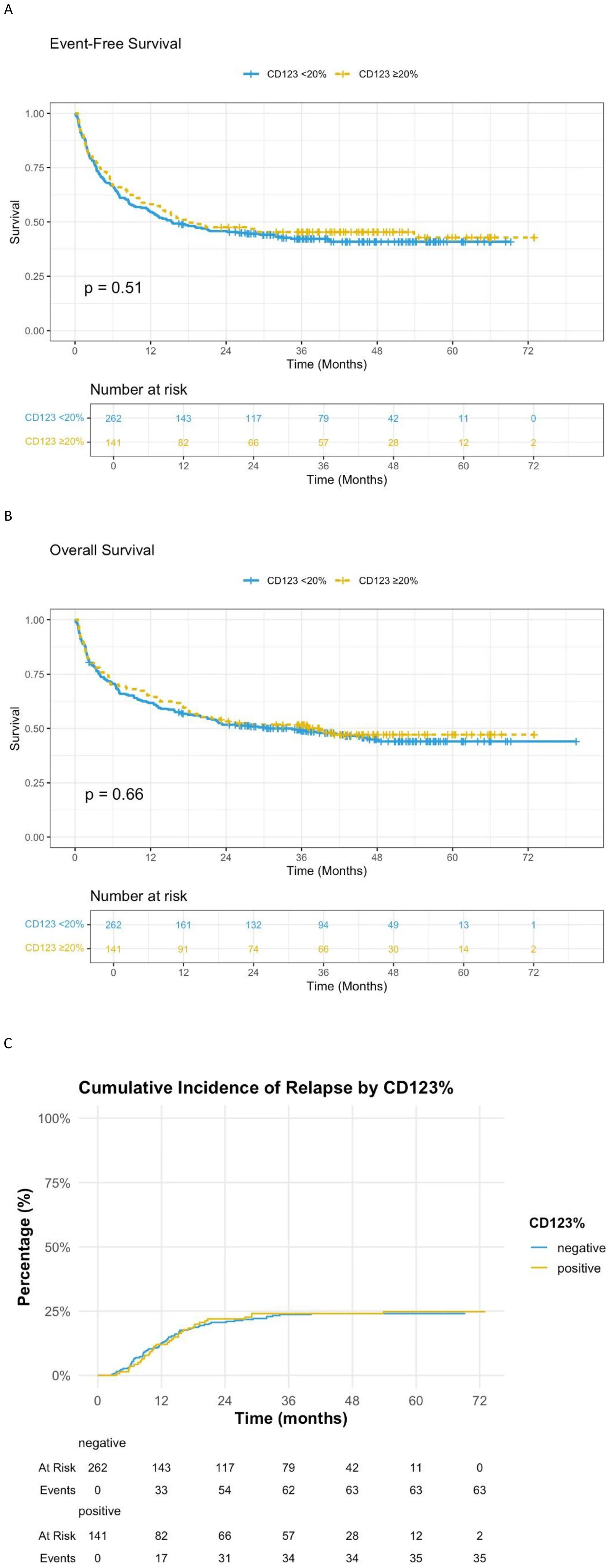
Survival analysis for the whole cohort according to expression of CD123 with cut-off point for positivity 20% **(A)**: Event-free survival (EFS), **(B)**: overall survival (OS), **(C)**: Cumulative incidence of relapse.

Three-year EFS, OS and CIR rates for the whole cohort were 43.3% (95% CI; 38.7-48.5), 50% (95% CI; 45.3-55.2) and 23.8% (95% CI; 19.8-28.1), respectively. The 3-year EFS was 45.3% (95% CI; 37.8-54.3) for CD123^+ve^ compared to 42.2% (95% CI; 36.6-48.7) in CD123^-ve^ patients (*P = 0.5*) and the 3-year OS was 51.7% (95% CI; 44.1-60.7) and 49% (95% CI; 43.2-55.5), respectively (*P = 0.6*). The cumulative incidence of relapse at 3 years for CD123^+ve^ was 24.1% (95% CI; 17.4-31.5), comparable to 23.7% (95% CI; 18.7-29) for CD123^-ve^ patients (*P = 0.9*) (**Figures 2A–C**). In addition, no statistically significant association was observed between median CD123 expression (46%) and either survival rates or CIR. Similarly, there were no statistically significant differences between CD123^+ve^ and CD123^-ve^ expression in relation to either survival rates or CIR among the risk groups or MRD post-induction II. The primary survival analysis is summarized in **Table 3**.

**Table 3:** Event-free survival (EFS), overall survival (OS), and cumulative incidence of relapse (CIR) according to CD123 expression in the overall cohort and stratified by initial risk group, final risk group, and post-induction II MRD status.

CD123 expression	3-year EFS% (95% CI)	P value*	3-year OS% (95% CI)	P value*	3-year CIR (95% CI)	P value*
For the whole cohort	43.3 (38.7-48.5)		50.0 (45.3-55.2)		23.8 (19.8-28.1)	
According to CD123% expression						
< 20%	42.2 (36.6-48.7)	0.51	49.0 (43.2-55.5)	0.66	23.7 (18.7-29.0)	0.9
≥ 20%	45.3 (37.8-54.3)		51.7 (44.1-60.70)		24.1 (17.4-31.5)	
According to median of CD123% expression						
< 46%	50.8 (39.4-65.6)	0.23	57.8 (46.3-72.2)	0.31	22.9 (12.9-34.6)	0.65
≥ 46%	41.5 (32.2-53.6)		47.6 (38.0-59.5)		25.1 (16.3-34.8)	
CD123% within the initial risk groups						
LR						
< 20%	62.7 (53.5-73.5)	1	66.9 (57.8-77.4)	0.97	12.1 (6.4-19.8)	0.56
≥ 20%	61.4 (48.5-77.6)		70.5 (58.2-85.3)		15.9 (6.9-28.3)	
IR						
< 20%	33.4 (26.4-42.3)	0.33	41.1 (33.7-50.2)	0.56	29.9 (22.5-37.6)	0.41
≥ 20%	42.3 (32.2-55.5)		46.5 (36.2-59.7)		23.9 (14.7-34.4)	
HR						
< 20%	19.3 (8.9-42.1)	0.4	30.2 (17.1-53.1)	0.44	31.7 (15.1-49.8)	0.57
≥ 20%	26.4 (13.8-50.6)		34.6 (20.4-58.7)		39.0 (19.9-57.8)	
CD123% within the final risk groups						
LR						
< 20%	62.5 (52.9-74.0)	0.78	68.8 (59.6-79.6)	0.7	12.4 (6.3-20.7)	0.37
≥ 20%	64.1 (50.7-81.1)		74.4 (61.8-89.4)		17.9 (7.8-31.5)	
HR						
< 20%	35.6 (28.9-43.9)	0.32	43.1 (36.0-51.6)	0.54	32.0 (24.9-39.2)	0.62
≥ 20%	41.9 (32.9-53.2)		47.3 (38.2-58.6)		29.0 (20.1-38.5)	
CD123% within MRD level post Induction II						
MRD <0.1%						
< 20%	53.1 (43.0-65.5)	0.11	64.2 (54.2-75.9)	0.11	30.9 (21.1-41.1)	0.73
≥ 20%	67.8 (55.9-82.1)		80.0 (69.6-91.9)		26.0 (14.7-38.8)	
MRD ≥0.1%						
< 20%	55.6 (47.4-65.4)	0.28	62.6 (54.5-71.9)	0.15	30.6 (22.6-39.0)	0.81
≥ 20%	46.7 (35.8-61.0)		51.5 (40.4-65.6)		33.9 (22.3-45.8)	

*Significant P-value < 0.05. LR, low risk; IR, intermediate risk; HR, high risk; MRD, minimal residual disease.

Of the 403 patients, 15 died within the first 14 days of diagnosis either before treatment initiation or from disease-related complications. To provide a more accurate assessment of treatment-related outcomes, a sensitivity analysis excluding these patients was performed, leaving 388 patients eligible for survival analysis. The findings remained consistent, with no significant differences in EFS, OS or CIR observed between the CD123^+ve^ and CD123^-ve^ groups (**Supplementary Table 1**).

Multivariable Cox regression analyses were performed to identify independent predictors of OS and EFS. CD123 expression was not independently associated with either OS (HR = 0.87, 95% CI: 0.65–1.18, *P* = 0.39) or EFS (HR = 0.85, 95% CI: 0.64–1.13, *P* = 0.28). CBF leukemia was an independent favorable prognostic factor for both OS and EFS (both *P* < 0.001), whereas monosomy 7 was independently associated with inferior OS (*P* = 0.04) and EFS (*P* = 0.03). HSCT, included as a time-dependent covariate, was independently associated with improved OS (*P* = 0.02), while its association with EFS did not reach statistical significance (*P* = 0.06). Age more than 10 years old, initial white blood cell count, FLT3 -ITD mutation, KMT2A rearrangement, and complex karyotype were not independently associated with OS or EFS (**Table 4**).

**Table 4:** Multivariate Cox regression analysis for event-free survival (EFS) and overall survival (OS).

Variable	EFS			OS		
	HR	95% CI	P value*	HR	95% CI	P value*
CD123	0.85	0.64-1.13	0.28	0.87	0.65-1.18	0.39
Age > 10 years	1.20	0.91-1.59	0.18	1.20	0.90-1.61	0.19
White blood cells	1.00	0.99-1.00	0.26	1.00	0.99-1.00	0.27
Core binding factor	0.46	0.32-0.65	<0.001	0.43	0.29-0.62	<0.001
FLT3-ITD	0.88	0.49-1.58	0.68	1.00	0.55-1.79	0.99
KMT2A	0.89	0.57-1.39	0.62	0.96	0.61-1.50	0.87
Monosomy 7	1.80	1.05-3.07	0.03	1.74	1.00-3.01	0.04
Complex karyotyping	1.69	0.98-2.91	0.056	1.41	0.77-2.58	0.25
Hematopoietic stem cell transplant (time-dependent)	0.25	0.06-1.07	0.06	0.10	0.01-0.78	0.02

*Significant P-value < 0.05. FLT3-ITD with allelic ratio >0.4. EFS, event free survival; OS, overall survival; HR, hazard ratio.

## 4 Discussion

CD123 has attracted increasing attention in AML for its role in leukemic stem cell biology, its association with high-risk disease phenotypes, and its relevance as a therapeutic target. In this comprehensive pediatric cohort of 403 *de novo* AML patients, we characterized CD123 expression in relation to clinical, biological, and outcome measures. Using MPFC and a cut-off of 20% for CD123 positivity, 35% of the patients exhibited overexpression of CD123. Previous adult AML studies reported diverse rates using similar thresholds, 39% in Jiang et al. and 97% in Al-Mawali et al. (20). A study conducted by Das et al.; including both pediatric and adult patients with AML, B-ALL, and T-ALL; found that CD123 expression in AML patients was observed in 75.6%, 66.2%, and 50% of cases when using thresholds of 5%, 10%, and 20%, respectively (12).

In our study, CD123 positivity had statistically significant association with higher WBC counts and BM blast percentages. These findings align with the work of Testa et al., who demonstrated that IL-3R (CD123) promotes proliferation of both normal and leukemic progenitors. In their study, patients with high IL-3R expression exhibited a significantly higher median WBC count (81 × 10^9^/L) compared with those expressing lower levels (21).

With respect to FAB classification, subtypes M4 and M5 in our study had significantly higher proportions of CD123^+ve^ expression than the other subtypes. This finding is consistent with the adult AML study conducted by Ehninger et al., which reported high CD123 expression in M3, M4, and M5 (22). This finding supports the association between monocytic-lineage AML and elevated CD123 expression, which may reflect the underlying biological and leukemia subtype characteristics.

Investigating genetic associations, a lower percentage of cases with t(8,21) and a higher percentage of *FLT3-ITD* had CD123^+ve^ expression. No significant association was observed in relation to *KMT2A-r*, Monosomy 7, or complex karyotype. These results align closely with a report from the COG that evaluated CD123 expression in a large pediatric AML cohort. CD123 levels were quantitatively measured using MPFC and the cohort was stratified into four quartiles based on their expression levels. Their study showed that the prevalence of t(8;21), inv (16), and *CEBPA* mutations was inversely associated with CD123 expression. However, *NPM1* mutations occurred at similar rates across all the CD123 expression quartiles. In contrast, among the genetically high-risk patients, a direct association was observed between CD123 expression and *FLT3-ITD*. No significant association was found between CD123 expression and monosomy 7 (19).

The differential expression across genetic risk groups, particularly the inverse association with favorable-risk t(8;21) and direct association with high-risk *FLT3-ITD*, may suggest its association with unfavorable prognostic features. Despite no significant association being observed in relation to *KMT2A-r*, Monosomy 7, or complex karyotype, the 35% incidence of CD123 expression reinforces its rationale as a therapeutic target for a subset of pediatric AML patients, who might benefit from CD123-targeted therapies possibly in combination with HSCT in the high-risk group.

Several CD123-targeted therapeutic strategies are currently under development, including antibody–drug conjugates, Tagraxofusp (a recombinant Fusion Protein, IL3-Diphtheria Toxin), CD123×CD3 bispecific T-cell engagers, and CD123-directed chimeric antigen receptor (CAR) T-cell therapies. Although these approaches have demonstrated promising preliminary antileukemic activity, their efficacy and safety remain under investigation, and none has yet been established as standard therapy for pediatric AML. Among these CD123 targets, Tagraxofusp is now approved by Food and Drug Administration (FDA) for BPDCN treatment in adults and pediatric patients two years and older. In addition, preclinical studies have demonstrated promising activity of tagraxofusp against AML and myelodysplastic syndromes (MDS), supporting the continued investigation of CD123-targeted therapies in these malignancies (23).

CD123 expression showed no significant association with treatment response at the end of induction I or II, whether assessed by BM morphology or MRD, consistent with COG findings (19). This contrasts with Das et al., who reported an association between high percentage of CD123 at cutoff ≥20% and MRD levels ≥0.1% after the end of induction II (12). This discrepancy could be attributed to differences in sample size, patient demographics, study population which included adult patients, defining and quantifying CD123 expression. Additionally, differences in treatment protocols and the timing or sensitivity of MRD assessments might have contributed to variations in the findings.

The three-year survival analysis revealed no significant differences in EFS, OS, or CIR in the studied cohort. Further stratification by initial and final risk groups and post-induction II MRD status consistently demonstrated that CD123 positivity was not associated with differences in survival outcomes. Notably, the survival outcomes were comparable between CD123^+ve^ and CD123^-ve^ patients; however, this observation was not sustained when survival was assessed using the median expression level of CD123 as a cutoff, which showed inferior survival for cases with median CD123 expression ≥ 46% compared to those with <46%, but was still not statistically significant. This suggests that the prognostic relevance of CD123 may be influenced more by its degree of expression than by a simple positive/negative classification. Higher levels of CD123 expression might reflect distinct biological behaviors that could affect outcomes, an effect that might be overlooked when broad thresholds are applied. These findings align with Jiang et al., who reported no significant association between CD123 positivity and survival outcomes but found that patients with high CD123 expression >72% had significantly shorter EFS and OS (11). In concordance, the COG study demonstrated that patients in the highest CD123 expression quartile had significantly worse survival and relapse risk, although high expression did not override favorable prognosis in low-risk cytogenetic groups (19).

Multivariable analysis did not identify CD123 expression as an independent prognostic factor. Despite its association with higher disease burden and the adverse FLT3-ITD, CD123^+ve^ was not associated with inferior survival outcomes, suggesting that it reflects underlying disease biology rather than serving as an independent predictor of clinical outcome.

Collectively, these data suggest that CD123 expression alone may not be a universal prognostic marker in pediatric AML; rather, its prognostic and therapeutic relevance likely depends on its interaction with specific genetic subgroups and expression levels. The primary limitation of this study is its retrospective, single-center design, which may introduce selection bias, incomplete or inconsistent data, limit generalizability to broader populations, and reduce the ability to control for confounding variables. Although the overall cohort numbers were adequate, subgroup analyses may have been underpowered to detect subtle associations. Additionally, we did not assess CD123 expression at the time of relapse, which precluded the evaluation of its stability over the course of the disease. While previous studies have suggested that CD123 expression remains stable at relapse, this needs to be confirmed in future prospective investigations.

## 5 Conclusions

CD123 has been overexpressed in nearly one third of pediatric AML patients with strong predilection for those with high-risk features including *FLT3-ITD* mutation and high initial disease burden, while lower levels were associated with favorable-risk aberrations t(8,21). CD123 overexpression at diagnosis did not demonstrate a significant association with early treatment response or survival outcomes. Although CD123 positivity may not confer prognostic implications, its positive expression in a considerable percentage of AML patients and its differential expression in leukemic cells versus normal hematopoietic cells have generated interest in its potential as a target for tailored therapies. Future prospective larger cohort studies are warranted to better assess subgroup-specific prognostic relevance of CD123 expression and to explore the impact of its quantitative levels rather than a simple positive/negative classification. Future investigations may assess CD123 expression at multiple time points (e.g., at diagnosis, post-induction, and relapse) to help elucidate its dynamic role during disease progression and treatment response, which may provide insights into its therapeutic potential beyond surface expression.

## Data Availability

The original contributions presented in the study are included in the article/**Supplementary Material**. Further inquiries can be directed to the corresponding authors.

## Supplementary material

Supplementary Table 1 Sensitivity Analysis of Event-free survival (EFS), overall survival (OS), and cumulative incidence of relapse (CIR) after excluding early deaths, according to CD123 expression in the overall cohort and stratified by initial risk group, final risk group, and post-induction II MRD status. *Significant P-value < 0.05. LR, low risk; IR, intermediate risk; HR, high risk; MRD, minimal residual disease.

Supplementary Figure 1 Roadmap for treatment adapted from COGAAML 1031.

Supplementary Figure 2 Dot plot histogram of gating strategy for CD123: **(A)** Forward Scatter (FSc) on X axis versus Side Scatter (SSc) on Y axis to identify the size and granularity of collected cells. **(B)** The second plot displays the antibody marker CD45 on X axis versus SSc on Y axis for gating of dim CD45 malignant clone. **(C)** Four-quadrant dot plot differentiating positive and negative populations depending on Isotype control plot analysis.

Supplementary Figure 3 Examples of histograms of CD123 expression at diagnosis. Green color represents the isotype control. Purple color represents CD123 expression. This histogram illustrates CD123 (PE) expression analyzed by flow cytometry, with fluorescence intensity displayed on a logarithmic scale (X-axis) and event count on the Y-axis. **(A-C)** The majority of cells fall within Gate G, with a relatively high geometric mean fluorescence intensity, indicating strong CD123 expression in most of the analyzed population. A smaller subset is included in Gate F, showing lower fluorescence intensity, consistent with low or negative CD123 expression**. (D)** The majority of events fall within Gate F, with a low geometric mean fluorescence intensity, indicating low or negative CD123 expression in most cells.
