# Supplementary material for: Prognostic impact of CD123 overexpression on the outcome of pediatric acute myeloid leukemia
Source: Front Oncol. 2026 Aug 21;16:1879782. doi: 10.3389/fonc.2026.1879782 (PMC13541458; doi:10.3389/fonc.2026.1879782)
Supplement: Supplementary file 1 [file Table1.docx]

**Supplementary table:**

**Supplementary table 1:** Sensitivity Analysis of Event-free survival (EFS), overall survival (OS), and cumulative incidence of relapse (CIR) after excluding early deaths, according to CD123 expression in the overall cohort and stratified by initial risk group, final risk group, and post-induction II MRD status

| Three-year Survival analysis | 3-year EFS %  (95% CI) | P value* | 3-year OS %  (95% CI) | P  value* | 3-years CIR  (95% CI) | P  value* |
| --- | --- | --- | --- | --- | --- | --- |
| For the whole cohort: | 45 (40.1-49.9) |  | 51.9 (46.8-56.9) |  | 25.1% (20.8-29.5) |  |
| According to CD123 % expression: | | | | | | |
| <20%  ≥20% | 44 (37.7-50.2)  46.6 (38.2-55.0) | 0.6 | 51.1 (44.8-57.3)  53.2 (44.7-61.6) | 0.7 | 25.3 (20-30.9)  24.9 (18-32.5) | 0.9 |
| According to median of CD123 % expression: | | | | | | |
| <46%  ≥46% | 52.7 (41-67.7)  42.5 (33.1-54.7) | 0.2 | 59.9 (48.3-74.4)  48.7 (39-60.8) | 0.2 | 23.7 (13.4-35.7)  29.6 (18.4-41.7) | 0.6 |
| CD123 % within the initial risk groups: | | | | | | |
| LR:  <20%  ≥20% | 64.1 (54.1-74)  62.8 (48.3-77.3) | 0.9 | 68.4 (58.6-68.2)  72.1 (58.7-85.4) | 0.9 | 12.5 (6.6-20.4)  16.3 (7-28.9) | 0.5 |
| IR:  <20%  ≥20% | 35.7 (27.5-43.9)  44.1 (32.3-55.8) | 0.3 | 44 (35.3-52.6)  48.5 (36.5-60.4) | 0.6 | 33.7 (25.5-42.1)  32.4 (16.3-49.5) | 0.3 |
| HR:  <20%  ≥20% | 19.3 (4.2-34.3)  26.4 (9.2-43.6) | 0.4 | 30.2 (13.1-47.2)  34.6 (16.3-52.8) | 0.4 | 32.4 (15.3-50.96)  39 (19.9-57.8) | 0.6 |
| CD123 % within the final risk groups: | | | | | | |
| LR:  <20%  ≥20% | 63.3 (52.7-73.8)  65.8 (50.7-80.8) | 0.6 | 69.7 (59.7-79.7)  76.3 (62.7-89.8) | 0.6 | 12.6 (6.4-21.1)  18.4 (8-32.3) | 0.3 |
| HR:  <20%  ≥20% | 36 (28.5-43.4)  42.3 (32.1-52.5) | 0.3 | 44.5 (36.6-52.3)  47.8 (37.6-57.9) | 0.5 | 33.9 (26.5-41.5)  34.1 (21.8-46.8) | 0.5 |
| CD123% within MRD level post Induction II: | | | | | | |
| MRD <0.1%  < 20%  ≥ 20% | 53.1 (43.0-65.5)  67.8 (55.9-82.1) | 0.1 | 64.2 (54.2-75.9)  80 (69.6-91.9) | 0.1 | 30.6 (22.6-41.1)  26.0 (14.7-38.8) | 0.7 |
| MRD ≥0.1%  < 20%  ≥ 20% | 55.6 (47.4-65.4)  46.7 (35.8-61.0) | 0.2 | 62.6 (54.5-71.9)  51.5 (40.4-65.6) | 0.1 | 30.6 (22.6-39.0)  33.9 (22.3-45.8) | 0.8 |

*Significant P-value < 0.05. Abbreviations: LR: low risk, IR: intermediate risk, HR: high risk. MRD: minimal residual disease
